# Virus taxonomy proposal summaries: a searchable and citable resource to disseminate virus taxonomy advances

**DOI:** 10.1099/jgv.0.002079

**Published:** 2025-07-25

**Authors:** Richard Mayne, Peter Simmonds, Donald B. Smith, Evelien M. Adriaenssens, Elliot J. Lefkowitz, Hanna M. Oksanen, Francisco Murilo Zerbini, Poliane Alfenas-Zerbini, Frank O Aylward, Juliana Freitas-Astúa, R. Curtis Hendrickson, Holly R. Hughes, Mart Krupovic, Jens H. Kuhn, Małgorzata Łobocka, Arcady R. Mushegian, Judit Penzes, Alejandro Reyes Muñoz, David L. Robertson, Simon Roux, Luisa Rubino, Sead Sabanadzovic, Nobuhiro Suzuki, Dann Turner, Koenraad Van Doorslaer, Arvind Varsani

**Affiliations:** 1 Nuffield Department of Medicine, University of Oxford, Peter Medawar Building, South Parks Road, Oxford OX1 3SY, UK; 2 Quadram Institute Bioscience, Norwich Research Park, Norwich NR4 7UQ, UK; 3 Department of Microbiology, University of Alabama at Birmingham, BBRB 276, 845 19th St South, Birmingham, AL 35294-2170, USA; 4 Molecular and Integrative Biosciences Research Programme, Faculty of Biological and Environmental Sciences, University of Helsinki, Viikinkaari 9, 00014 Helsinki, Finland; 5 Departamento de Fitopatologia/BIOAGRO, Universidade Federal de Viçosa, Viçosa, MG 36570-900, Brazil; 6 Departamento de Microbiologia, Universidade Federal de Viçosa, Viçosa, MG 36570-900, Brazil; 7 Department of Biological Sciences, Virginia Tech, Blacksburg, VA, USA; 8 Embrapa Cassava and Fruits, Cruz das Almas, Bahia, 44380-000, Brazil; 9 Centers for Disease Control and Prevention, Fort Collins, Colorado, USA; 10 Institut Pasteur, Université Paris Cité, CNRS UMR6047, Archaeal Virology Unit, 25 rue du Dr Roux, 75015 Paris, France; 11 Integrated Research Facility at Fort Detrick, National Institute of Allergy and Infectious Diseases, National Institutes of Health, B-8200 Research Plaza, Fort Detrick, Frederick, MD 21702, USA; 12 Institute of Biochemistry and Biophysics of the Polish Academy of Sciences, 02-106 Warsaw, Poland; 13 Division of Molecular and Cellular Biosciences, National Science Foundation, 2415 Eisenhower Avenue, Alexandria, VA 22314, USA; 14 Institute for Quantitative Biomedicine, Rutgers University, Piscataway, New Jersey, USA; 15 Departamento de Ciencias Biológicas, Universidad de los Andes, Bogotá, Colombia; 16 MRC-University of Glasgow Centre for Virus Research, Sir Michael Stoker Building, 464 Bearsden Road, Glasgow G61 1QH, UK; 17 Department of Energy, Joint Genome Institute, Lawrence Berkeley National Laboratory, Berkeley, California, USA; 18 Consiglio Nazionale delle Ricerche, Istituto per la Protezione Sostenibile delle Piante, Sede Secondaria di Bari, Via Amendola 165/A, 70126 Bari, Italy; 19 Department of Agricultural Science and Plant Protection, Mississippi State University, Mississippi State, MS 39762, USA; 20 Institute of Plant Science and Resources, Okayama University, Kurashiki, Okayama 710-0046, Japan; 21 Molecular Biology, University of the West of England, Bristol, UK; 22 Department of Immunobiology, School of Animal and Comparative Biomedical Sciences, BIO5 Institute, University of Arizona Cancer Center, Tucson, AZ 85721, USA; 23 The Biodesign Center for Fundamental and Applied Microbiomics, School of Life Sciences, Center for Evolution and Medicine, Arizona State University, Tempe, AZ 85287-4701, USA

**Keywords:** ICTV, master species list, taxonomy proposal, virus taxonomy

## Abstract

Taxonomic classification of cellular organisms requires the publication of descriptions and proposed names of species and the deposition of specimens. Virus taxonomy is developed through a different system of annual submission of formal taxonomy proposals (TPs) that can be submitted by anyone but are typically prepared by a study group appointed by the International Committee on Taxonomy of Viruses (ICTV) and consisting of experts on a particular group of viruses. These are initially evaluated by an expert subcommittee and by the executive committee (EC) of the ICTV. EC-approved TPs are then submitted for evaluation and a ratification vote by the wider ICTV membership. Following ratification, the new taxonomy is annually updated in the Master Species List, associated databases and bioinformatic resources. The process is consistent, creates traceability in assignments and supports a fully evaluated, hierarchical classification and nomenclature of all taxonomic ranks from species to realms. The structure also facilitates large-scale and coordinated changes to virus taxonomy, such as the recent introduction of a binomial species nomenclature.

TPs are available on the ICTV website after ratification, but they are not indexed in bibliographic databases and are not easily cited. Authors of TPs do not receive citation credit for adopted proposals, and their voluntary contributions are largely invisible in the published literature. For greater visibility of TPs and their authors, the ICTV will commence the annual publication of summaries of all TPs from each ICTV subcommittee. These summaries will provide a searchable compendium of all annual taxonomy changes and additions as well as direct links to the Master Species List and other ICTV bioinformatic resources. Their publication will provide due credit and citations for their authors, form the basis for disseminating taxonomy decisions and promote greater visibility and accessibility to taxonomy changes for the virology community.

## Introduction

Taxonomy is an area of systematics that provides internationally agreed-upon classification and nomenclature frameworks for animals, plants, fungi, protists, prokaryotes and viruses. A universal taxonomy is essential for scientific communication, providing reference points for studies of the evolution and ecology of organisms. Taxonomy also informs regulatory frameworks for agricultural and livestock trade, biosecurity, medicine and public health.

## Taxonomic codes

In view of the importance of agreed-upon and universally used taxonomies, there has been considerable worldwide and cross-disciplinary effort to develop consistent rules for taxonomic assignments and to maintain internationally shared taxon names for classified organisms. Reflecting this need, the nomenclature of animals, plants, fungi, protists, prokaryotes (bacteria and archaea) and viruses has been coordinated and regulated by longstanding expert committees over the previous century or longer; the International Commission on Zoological Nomenclature (ICZN) produces the International Code of Zoological Nomenclature (https://www.iczn.org/the-code/the-code-online/); the International Association for Plant Taxonomy produces the International Code of Nomenclature for algae, fungi and plants (ICNafp; https://www.iaptglobal.org/icn); and the International Committee on Systematics of Prokaryotes produces the International Code of Nomenclature of Prokaryotes (ICNP [1]). For viruses, the International Committee on Nomenclature of Viruses, renamed to the International Committee on Taxonomy of Viruses (ICTV) in 1973, was commissioned in 1966 by what is now the International Union of Microbiological Societies to develop and maintain a universal virus taxonomy. The ICTV has since published and regularly updates the International Code of Virus Classification and Nomenclature (ICVCN; https://ictv.global/about/code).

With the exception of the ICVCN for viruses, these codes primarily regulate and recommend procedures for the formal description of species, the assignment of scientific names and the associated deposition of supporting material in international repositories. There are broad similarities among codes in the conventions used to create Latinized binomial scientific names that perpetuate a nomenclatural system first developed by Linnaeus in the eighteenth century [2]. Orthography remains largely based on mediaeval Latin grammar, and it is remarkably unchanged in its formatting and rules for word formation and declension. Codes for cellular organisms differ from each other in detail, such as numbering and naming conventions for below-species ranks, whether genus and species epithets can be identical [tautonyms; e.g. *Gorilla gorilla;* Savage, 1847 and indeed the subspecies *G. gorilla gorilla* (Western lowland gorilla) in the ICZN], the extent to which assignments at higher taxonomic ranks are supported, and in the number and type of available secondary taxon ranks. There are also differences among codes in how species may be formally described, how authority is formatted after the scientific name (for example, *Sclerophrys capensis* Tschudi, 1838, with variants of this format reflecting taxonomic histories, ranks and codes), how and where descriptions and nomenclature proposals are published and the requirement and nature of materials required to support a species proposal. For example, descriptions of bacterial and archaeal species are generally published in the International Journal of Systematic and Evolutionary Microbiology, which also publishes the ICNP code. There is no journal requirement for the publication of zoological and botanical species names and descriptions, the only criterion is that they are made publicly available in journal or book form.

There are many published compendia and online databases of classified species and proposed higher taxonomic ranks. For example, bacterial species are provided in an online database at https://lpsn.dsmz.de, and species of animals are listed in ZooBank (https://zoobank.org). The ICTV similarly maintains the Master Species List (https://ictv.global/msl) and associated metadata for each classified species of virus (https://ictv.global/vmr).

Collectively, the application of these codes for cellular organisms has provided biologists with comprehensive and relatively coordinated inventories of agreed-upon species names and taxonomic frameworks that broadly fulfil the requirements of the various stakeholders in biological, evolutionary, clinical and regulatory fields. The use of scientific names to specify international trade restrictions on defined organisms in the Convention on International Trade in Endangered Species of Wild Fauna and Flora regulations (https://cites.org/eng/app/appendices.php) exemplifies how biological nomenclature provides precision and authority to regulate the international movement of vertebrate and invertebrate animals and of plants.

## Alternative codes

Current taxonomies of cellular organisms inherit a 300-hundred-year-long historical legacy with substantial organizational and procedural baggage arising from classification systems that predate modern scientific publishing, online databases and genome sequencing technologies. Inference of genetic relatedness is better able to reconstruct evolutionary histories independently of phenotypic properties that guided the original classification of animals, plants, bacteria and viruses and may provide a firmer basis for a robust taxonomy, particularly at higher ranks. Proposals for a change towards a purely genomics-based classification of organisms include the International Code of Phylogenetic Nomenclature (PhyloCode) developed by the International Society for Phylogenetic Nomenclature (discussed in [3]). This proposes a purely cladistic classification based on metrics of sequence similarity that might better reconstruct an organism’s evolutionary history, provide a more transparent and objective classification of organisms (particularly at high taxonomic ranks) and provide the means to resolve the numerous examples of paraphyly and nomenclatural confusion (such as *Escherichia coli* and *Shigella* spp.) of currently classified species. Another taxonomic framework, SeqCode, proposes that genome sequences can be used for valid publication of names of prokaryotes, avoiding the requirement for culturability and deposition of type materials [4]. Sequence-based assignments may vastly expand the number and range of (genotypically defined) species that could be assigned in the future, particularly for what may amount to over a million species of non-cultivated bacteria and archaea that are currently excluded from prokaryotic taxonomy. Unified databases, including the Catalogue of Life (www.catalogueoflife.org) and Encyclopaedia of Life (https://eol.org), seek to catalogue the over 2 million currently classified biological species into a combined database that would break down the current organizational divisions between the zoological, botanical and microbiological taxonomy codes and databases.

## Taxonomy of viruses

Since its inception in 1966, the ICTV has developed and maintained a classification and nomenclature framework for viruses. At the outset, the ICTV considers viruses to be equivalently classifiable as cellular life. There are equivalences in taxonomic assignments of viruses with those in other codes. The virus taxonomy code, ICVCN, does, however, have to contend with the fact that viruses originated *de novo* multiple times during the evolution of cellular life, and the highest taxonomic rank, realm, has been devised as best as possible to assign viruses to what are deduced to be separate origin groups [5,6]. Below this rank, viruses have been classified into seven principal ranks (kingdom, phylum, class, order, family, genus and species) using a uniform and universal orthography, including rank-specific suffixes (such as -*viricetes* and -*virales* for class and order, respectively) generally resembling those of other codes. Recently, as a result of years of work by the ICTV in consultation with the virology community, the virus code has adopted and universally applied a binomial name format for species [5,7] comprising a genus name+species epithet, although without the compulsory Latinization of terms used in other codes. The genus name bears a -*virus* suffix, but the species epithet is ‘freeform’ (although restricted to the 26 letters of the mediaeval Latin alphabet, hyphens and numbers).

Virus taxonomy has more recently embraced evolutionary systematics as the basis for classification, permitting the assignment of species and higher ranks primarily based on metrics of genetic relatedness, often independently of phenotypic characterization or descriptions [8,9]. Accordingly, species can be assigned in the absence of a specimen or isolate using coding-complete genome sequences, deposited in one of the International Nucleotide Sequence Database Collaboration databases to provide a unique exemplar equivalent to a type specimen of other taxonomic codes. Viruses known only from their genomic sequences, such as those characterized in metagenomic analyses of environmental samples, can therefore be assigned taxonomically [10]; this has paved the way for a fivefold expansion in classified virus species over the last 5 years [11].

## ICTV organization

The ICTV regulates both the assignment of viruses and virus-like agents at all taxonomic ranks (from species to realm) and the nomenclature of these taxa. This contrasts with other biological taxonomies where the remit of official bodies is limited to the regulation of taxonomic names, rather than having the specific focus and regulatory role in the creation and recording of scientific names for species. Moreover, with viruses, there is no equivalent of the publication-and-attribution model for describing and naming new species that is followed throughout the rest of biology. Instead, changes and additions to virus taxonomy are initiated by formal taxonomy proposals (TPs), submitted annually to the ICTV EC by ICTV-associated expert study groups or by members of the virology community. TPs are publicly posted on the ICTV website before being initially reviewed by EC members and, after necessary modification, voted on and ratified by the wider ICTV membership that includes EC members, subcommittee (SC) and study group chairs, life members and national representatives. After ratification, taxonomy changes are implemented in the Master Species List, which serves as the primary and authoritative record of virus classification.

This decision-making process is an effective framework for managing biological classification. Proposals receive expert scrutiny before ratification and adoption to ensure compliance with nomenclature conventions and the use of declared and approved taxon assignment criteria, such as metrics of sequence similarity for species or other rank assignments. Problems, such as the existence of homonyms and disputes over precedence, cannot arise in virus classification. The ICTV also possesses the organizational structure to perform extensive coordinated changes to taxonomy, such as the formal adoption of a 15-rank hierarchy in 2017 [12] and the renaming of species to conform to a binomial format in 2023–2024 [5,13, 14]; such changes would be difficult and slow to coordinate in other taxonomy systems.

However, the development of virus taxonomy through the submission and ratification of TPs does not create a searchable published record of the taxonomy changes in bibliographic databases. This is despite the considerable scientific effort of authors to analyse data, prepare and write the formal proposals and revise them in light of the ICTV feedback – activities often equalling that of preparing manuscripts for journal publication. Furthermore, there are often no directly citable sources for formal taxonomic changes. For example, if someone wanted to cite the origin of the current name of the species for hepatitis C virus, *Hepacivirus hominis*, this could only be done by reference to a TP file stored on the ICTV website (in this case, in the proposal 2022.007S.Flaviviridae_1genren_sprenamed) rather than by reference to a specific publication. Even much broader changes, such as the creation of higher taxonomic ranks or the introduction of binomial species names, lack defined publications that describe the specific changes actually made by the ICTV. With some notable exceptions, such as the annual publication of taxonomy changes to RNA viruses in the phylum *Negarnaviricota* [15,16], proposers and authors of TPs, who are the key constituency in the advancement of virus classification, generally receive no published acknowledgement or citations for their contributions towards what may be major changes and additions to taxonomy. The consequent ‘invisibility’ in the published literature may thus disincentivize many virologists from actively contributing to virus taxonomy by preparing TPs and greatly hamper individuals from finding and appropriately citing sources for taxonomy statements.

## A publication strategy for ratified virus TPs

To address these issues, The ICTV will publish taxonomy advances in the form of summaries of ratified virus TPs. These summaries are intended to be generated annually for all accepted proposals from each of the seven currently existing ICTV SCs and for general proposals immediately following the ratification vote. This will create a total of eight citable publications per year, each with a DOI, in the *Journal of General Virology*, a partner journal of the ICTV.

A pipeline has been developed for the automated extraction of proposal abstracts, the creation of tables itemizing the taxonomy changes and the creation of combined author and taxon keyword lists (Appendix I, available in the online Supplementary Material) that will greatly facilitate the production and ensure the accuracy of the summaries. Automation of this process is only possible because the submission of TPs to the ICTV follows a set of procedures involving strictly formatted documents from which the necessary information can be reliably extracted.

### Format of summary

**Fig. 1.**
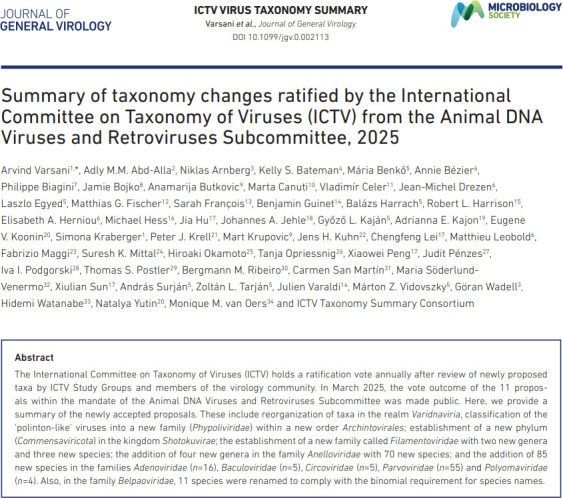
Example of title page listing title, all contributing authors and their affiliations.

**Fig. 2.**
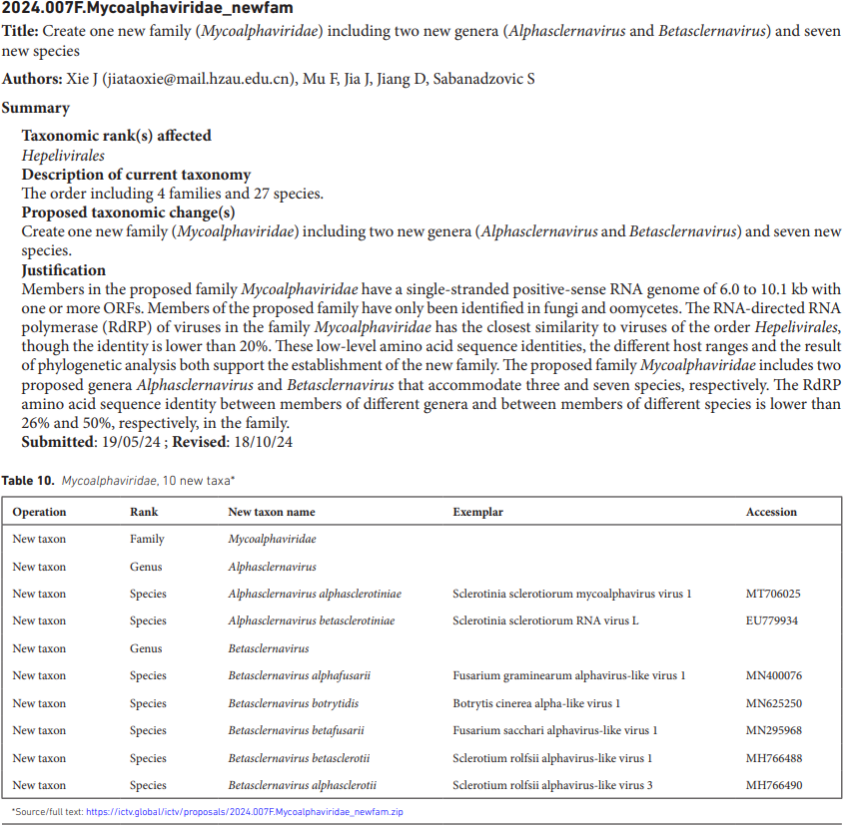
Example of an individual TP within the summary.

A summary will contain the following elements:

- **Title** (Fig. 1). This follows a standard format ‘Summary of taxonomy changes ratified by the International Committee on Taxonomy of Viruses (ICTV) from the {subcommittee name}, {year}’.
- **Author list and affiliations**. This is a compendium of all authors who contributed ratified proposals to the SC, listed in alphabetical order following the SC chair as the first author. The affiliations of all authors are ordered to correspond to the author list.
- **Abstract**. This is a general summary of the main taxonomic developments described within the summaries, written by the SC chair.
- **Introduction**. This is a variable-length section that might include the number of taxonomy additions and changes (e.g. the number of species and higher taxa established) as well as broader information on changes of perceived significance for the field in taxon assignments or methodologies used.
- **Content list**. This indexes the TPs in a tabulated list of the ICTV codes and titles of each ratified TP included in the document, with hyperlinks to the positions of the corresponding sections of the document.
- **Individual summaries** (Fig. 2). Each ratified TP is listed separately and sequentially. Each of these consists of
  - The unique TP code assigned by the ICTV to the proposal;
  - TP title;
  - Proposal authors, including the corresponding author/s as indicated by an email address;
  - Summary;
  - Submission and revision dates;
  - Tabular summary of the taxonomy additions and changes; and
  - Source data, a hyperlink to the full TP documents on the ICTV website (indexed under https://ictv.global/files/proposals/approved).
- **Keywords**. This is an alphabetically sorted list of new or changed taxon names referred to in the summaries, including previously used terms for taxa that have been renamed or abolished. The keyword list provides indices for searches in PubMed and other bibliographic sources that enable taxa to be found and cited in general literature searching.
- **References** to papers cited in the Introduction.
- **Master table**. A master table will be included as a supplementary Excel file, containing a formatted list of all tabular data for a TP summary. All data are stored in one sheet and are identical to their original representation in the TP summary, with the exception of an additional column to indicate from which TP they originate.

### Citing a taxonomy change

The summaries are designed to provide a published and citeable source for all taxonomic changes such as the assignment of new taxa or re-classifying or renaming virus taxa. We propose that citations of the summary should be supplemented with the relevant ICTV taxonomy proposal code in the format

"…. and a new family for Nitrosopumilaceae virus NYM1 [34] (Yimin *et al.*; proposal 2024.003A)"

where [34] is the citation for the Taxonomy Summary, “Yimin *et al*.“ is an optional reference to the authorship of the proposal and “2024.003A” is the abbreviated ICTV code for the specific taxonomy proposal within the cited summary.

Reference to multiple proposals within a summary could be formatted as in this example:

“Two additional species of orthohantaviruses were described [34] (proposals 2024.001M and 2024.017M).”

## Conclusions

The creation of annual virus taxonomy summaries for the seven ICTV SCs and of general proposals represents an entirely new strategy for the publication of virus taxonomy changes. This approach is quite different in scope and authority from the less-regulated publication of species descriptions and nomenclature followed by other taxonomic codes. The virus taxonomy summaries will provide author attributions, searchable content and linkage to ICTV taxonomy proposals and Master Species List databases. We believe that the virus taxonomy summaries will be effective in disseminating and providing a citable source for virus taxonomy information in the future.

## Appendix I: a pipeline for the generation of TP summaries

Input data to the TP summary system comprise paired TP documents, currently in the form of a “Word module” (WM) and an ‘Excel module’ (EM), both of which have undergone recent revisions to better permit their being parsed. The summary system comprises three modules:

1. Tabular data parser, which extracts and structures taxonomic ranks from the EM;
2. Text parser, which extracts, cleans and interprets supporting information from the WM; and
3. Report generator, which takes data generated from tabular and text parser modules and creates output documents.

The parser components of the summary system extract all data from both WM and EM, although not all data from the former are structured into TP summary documents. A test suite is built into the system, such that many common invalid responses by TP authors are flagged and, in some simple cases, corrected. Examples of such errors include missing fields, mis-pairings of authors to their affiliation addresses and inclusion of submission dates in a valid but incorrect format; the latter may be automatically corrected. Depending on the severity of an error, the test suite may either cause a breakpoint in the code, requiring the user’s attention, or may collect errors in a report for later correction.

The summary system is written in Python 3.10 and relies on Pandas (https://pandas.pydata.org/) and PyDocX (https://github.com/CenterForOpenScience/pydocx) libraries to parse input files and generate output. The three modules are packaged as an application programming interface (API) via FastAPI (https://fastapi.tiangolo.com/), designed to act as a stand-alone application from a Docker container such that it can be run on any operating system.

The software parses all of the files in a user-specified folder and generates a summary document. This is designed for use at the end of ICTV ratification rounds, with the intention of creating a publishable reference source for the year’s TPs. Summaries will be created for all of the TPs within an SC each year.

The main output, hereafter ‘summary documents’, are Microsoft Word files containing summarized TP data from both EMs and WMs. Accompanying tabular files are generated, containing the author list and ‘master tables’, which contain all of the summarized EMs in a single sheet.

To test the summary system, TP summaries were generated for each of the six ICTV SCs with the 109 TPs from the 2025 ratification round. (There were no animal+ssRNA (S) TPs during this period.) Then, TP summaries were manually checked by the ICTV Executive Committee and development team, which iteratively refined the summary system.

### Performance

In cases for which no errors were identified in the underlying data, TP summaries were generated in ~5 s on a standard consumer-grade laptop. Of the 109 individual TPs, the summary system identified 66 WM documents as having errors, of which 46 were human errors and 20 were due to incompatibilities in language pack encoding. All WM errors were manually corrected. Only one error was identified in EMs, in which a title field was not present, which was also corrected manually. In total, all six summary documents were generated, checked and disseminated within a single day.

For future work, it will be additionally possible to use the summary system to parse TPs at the point of importation into the ICTV website. The benefit would be the automatic proofing of incoming submissions.

The source code is freely available on the GPL-3.0 licence at https://github.com/Mayne941/parse_taxonomy_proposal_form

## Abbreviations

API: application programming interface
EC: Executive Committee
EM: Excel module
ICNP: International Code of Nomenclature of Prokaryotes
ICTV: International Committee on Taxonomy of Viruses
ICVCN: International Code of Virus Classification and Nomenclature
ICZN: International Commission on Zoological Nomenclature
SC: subcommittee
TPs: taxonomy proposals
WM: Word module
